# Bridging bench to bedside: a dual-use framework for chromene-based anti-obesity and antiviral therapeutics

**DOI:** 10.3389/fphar.2025.1666414

**Published:** 2025-11-13

**Authors:** Sigrit Karla Chávez-Jiménez, Carlos Josué Solórzano-Mata, Beatriz Xóchitl Ávila-Curiel, Efrén Emmanuel Jarquín González, Edgar Zenteno, Hector A. Cabrera-Fuentes

**Affiliations:** 1 Laboratorio de Bioquímica de Proteínas y Glicopatologías, Faculty of Dentistry, Universidad Autónoma “Benito Juárez” de Oaxaca, Oaxaca, Mexico; 2 UNAM–UABJO Research Center, Faculty of Medicine and Surgery, Universidad Autónoma “Benito Juárez“ de Oaxaca, Oaxaca, Mexico; 3 Dirección General de los Servicios de Salud de Oaxaca, Secretaria de Salud, Servicios de Salud de Oaxaca, Oaxaca, Mexico; 4 Departamento de Bioquímica, Facultad de Medicina, Universidad Nacional Autónoma de México (UNAM), Mexico, Mexico; 5 División de Estudios de Posgrado e Investigación, Tecnológico Nacional de México, Instituto Tecnológico de Tijuana, Tijuana, Mexico; 6 R&D group, Vice Presidency Scientific Research and Innovation, Imam Abdulrahman bin Faisal University (IAU), Dammam, Saudi Arabia; 7 Dirección de la División de Investigación y Desarrollo Científico, Benemérita Universidad de Oaxaca, Oaxaca, Mexico

**Keywords:** synthetic chromenes, anti-obesity agents, green chemistry metrics, adipogenesis, structure–activity relationship (SAR), human immunodeficiency virus (HIV), Absorption, Distribution, Metabolism, Excretion, and Toxicity (ADMET) profiling, pharmacokinetics

## Introduction

Obesity afflicts more than 650 million adults worldwide and contributes to a wide range of cardiometabolic disorders, including type 2 diabetes, nonalcoholic fatty liver disease, and certain cancers (Boutari and Mantzoros, 2022; Abad-Jimenez and Vezza, 2025). Despite this burden, safe and orally available small-molecule therapies remain scarce, which has fueled growing interest in both natural and synthetic compounds capable of modulating adipogenesis (Zhao et al., 2022).

Recent work by Inthanon et al. (2025) has highlighted the anti-adipogenic and lipid-lowering properties of synthetic chromene derivatives in 3T3-L1 preadipocytes. These findings are consistent with the work of Cho et al. (2018), who characterized rotenoisin A, a novel tetrahydrochromene-based flavonoid, as a potent anti-adipogenic agent. Its activity was shown to involve activation of AMP-activated protein kinase (AMPK) and downregulation of key adipogenic transcription factors, including CCAAT/enhancer-binding protein alpha (C/EBPα) and peroxisome proliferator-activated receptor gamma (PPARγ) (Ahmad et al., 2020; Rosen et al., 2002).

Chromenes and their derivatives are oxygen-containing heterocycles present in many bioactive natural products, including flavonoids, coumarins and xanthones (Gaspar et al., 2024). Khalilpourfarshbafi et al. (2019) has studied the role of flavonoids such as cyanidin, rutin, naringenin, hesperidin, quercetin, naringin, and resveratrol as inhibitors of adipogenesis by suppressing factors such as CCAAT/enhancer-binding protein β (C/EBPβ) and PPARγ. Findings from these studies indicate that chromenes exert their biological activity through multiple, overlapping mechanisms, particularly by modulating antioxidant defenses, dampening inflammatory responses, and influencing key metabolic regulatory pathways.

Moreover, the therapeutic implications of chromene derivatives may extend beyond metabolic disorders. Several chromene-based molecules have demonstrated antiviral activity, most notably against human immunodeficiency virus (HIV) as reported by Inthanon et al. (2025) and against dengue virus as described by Dharmapalan et al. (2022), through inhibition of viral enzymes and suppression of viral replication. This dual action opens the possibility of repositioning chromenes in clinical scenarios characterized by coexisting metabolic and infectious diseases, such as HIV-associated lipodystrophy, where adipose tissue homeostasis and antiviral responses are simultaneously compromised.

Based on these findings, we propose a translational framework to advance synthetic chromenes from *in vitro* hits to clinical candidates. Our approach hinges on five pillars: rigorous green-chemistry validation, extended multi-phase biological evaluation (both *in vitro* and *in vivo*), deep mechanistic insight through structure–activity relationships (SAR) and molecular modeling, early absorption, distribution, metabolism, excretion, and toxicity (ADMET) profiling, and exploration of dual anti-obesity and antiviral potential particularly relevant for HIV-infected patients prone to lipodystrophy.

### Sustainable chemistry and translational evaluation

A fundamental pillar in sustainable drug development is the rigorous quantification of environmental impact (Wang et al., 2023). In the study conducted by Inthanon et al., the use of green synthesis methods is highlighted, representing a significant step toward this goal. To strengthen the environmental rigor of the study and enable cross-comparison of synthetic routes, we propose the inclusion of key sustainability metrics for the processes employed. Specifically, we recommend reporting quantitative parameters such as Atom Economy and E-Factor for each step, with target values of greater than 80% and less than 10 kg of waste per kilogram of product, respectively, while also applying the Green Analytical Procedure Index (GAPI) to identify “red zones” in reagent selection or energy consumption (Ahmed-Anwar et al., 2023; Płotka-Wasylka, 2018). For example, substitution of carbon-deuterated chloroform (CDCl_3_)—prone to decomposition into phosgene (Nazarski, 2023)—with aqueous ethanol or dimethyl sulfoxide (DMSO) has been shown to yield chromene products of >95% purity, improved crystallinity, and up to 60% less solvent waste (Jiang et al., 2020). Table 1 contrasts hypothetical green metrics across three synthesis routes, underscoring how a shift to EtOH/H_2_O can dramatically reduce environmental burden without compromising yield.

**TABLE 1:** Comparative green metrics for chromene syntheses.

Metric	CDCl_3_ route	Aqueous EtOH route	DMSO route	Target	Rationale
Atom economy (%)*	68	83	77	>80	Indicates the proportion of reactant mass incorporated into the final product; higher values minimize waste
E-Factor (kg/kg)**	25	5	8	<10	Mass of waste generated per kg of product; lower values reflect more efficient, less-polluting syntheses
GAPI score (red zones)***	3/5	1/5	2/5	≤2/5	Green analytical procedure index flags unsustainable steps; fewer red zones denote greener processes

^*^Atom Economy > 80% ensures most of the starting materials end up in the desired chromene product. **E-Factor < 10 kg/kg aligns with benchmarks for laboratory–scale pharmaceutical synthesis. ***GAPI, Score ≤ 2/5 restricts the number of “red” (high-impact) parameters, promoting overall sustainability.

In parallel, to capture the full complexity of adipogenesis, a time profile that goes beyond the standard 72-h interval is required. Therefore, as a complement to standard approaches, an extended time profile that matches the different biological phases of adipocyte development may be useful. Adipocyte formation unfolds in three distinct phases: commitment (days 0–3), differentiation (days 4–7), and maturation (days 8–10), each governed by coordinated transcriptional programs (Mukherjee et al., 2024; Mussbacher et al., 2020; Pazos et al., 2002). Early assays should quantify preadipocyte viability and C/EBPβ mRNA induction; mid-phase analyses must measure PPARγ and C/EBPα protein levels alongside nascent lipid droplet visualization; and late-stage evaluations ought to assess mature adipocyte biomarkers such as adiponectin and leptin secretion. We further recommend complementing *in vitro* findings with *in vivo* models, including diet-induced obesity (DIO) in C57BL/6J mice for whole-body metabolic readouts; Nile Red–stained zebrafish larvae for high-throughput screening of lipid deposition; and leptin-deficient (ob/ob) mice to interrogate effects on hepatic steatosis and dyslipidemia. Table 2 summarizes this multi-phase, cross-species evaluation pipeline. Mechanistic understanding is equally critical. We propose a systematic SAR campaign in which >25 chromene analogs bearing diverse substituents at positions 6 and 8 are screened for anti-adipogenic potency (Buettner et al., 2007; Kaczmarek et al., 2024; Liu et al., 2018; Tang et al., 2022; Zhang et al., 1994). Preliminary observations suggest that electron-withdrawing substituents at position C6 may facilitate cellular uptake, whereas C-8 methoxy substitutions improve receptor affinity (Yoshimori and Bajorath, 2025). Complementing SAR, molecular docking against the ligand-binding domains of PPARγ and C/EBPα as well as the regulatory domain of Sterol Regulatory Element Binding Protein 1c (SREBP1c), will predict binding modes and identify key interactions. High-scoring compounds should advance to 100-ns molecular dynamics simulations to validate complex stability, with *in silico* binding energies correlated against measured IC_50_ values for adipogenesis inhibition. This integrated in silico–in vitro pipeline accelerates lead optimization while minimizing resource consumption (Noreen et al., 2025).

**TABLE 2:** Integrated evaluation pipeline with rationale and success criteria*.

Model/Phase	Timing	Key endpoints	Methods	Rationale	Success Criteria (vs. Vehicle)
*In vitro*					
Early (Commitment) (Kaczmarek et al., 2024)	Days 0–3	Cell viability; C/EBPβ mRNA	MTT assay; qPCR Oil Red O staining; Differential Interference Contrast (DIC) microscopy	Captures preadipocyte health and onset of adipogenic transcription	≥90% viability; ≥50% C/EBPβ downregulation
Mid (Differentiation) (Kaczmarek et al., 2024)	Days 4–7	PPARγ and C/EBPα protein; lipid droplet formation	Western blot Oil Red O staining DIC microscopy	Measures master regulator expression and initial lipid accumulation	≥40% reduction in PPARγ/C/EBPα; ≥60% lipid decrease
Late (Maturation) (Kaczmarek et al., 2024; Tang et al., 2022)	Days 8–10	Adiponectin and leptin secretion	Immunocytochemistry; ELISA DIC microscopy	Assesses mature adipocyte function and endocrine output	≥30% increase in adiponectin and leptin secretion
*In vivo*					
Diet induced obese (DIO) Mice (Buettner et al., 2007)	12 weeks	Body weight; fat mass; glucose and insulin tolerance	MRI; metabolic cages; Glucose Tolerance Test (GTT) / Insulin Tolerance Test (ITT)	Reflects compound efficacy in diet-induced obesity and systemic metabolism	≥15% less body-weight gain; ≥20% fat-mass reduction; ≥25% improved GTT Area Under the Curve (AUC)
Zebrafish Larvae (Liu et al., 2018)	5 days post-fertilization	Whole-body lipid deposition	Nile Red fluorescence imaging	Provides high-throughput, cost-effective screening of lipid-lowering activity	≥50% reduction in Nile Red fluorescence
ob/ob Mice (Zhang et al., 1994)	4 weeks	Hepatic steatosis; plasma triglycerides and cholesterol	Histology; Liquid Chromatography–Mass Spectrometry (LC-MS) lipidomics	Evaluates efficacy in genetic obesity and lipid-storage disorders	≥30% fewer hepatic lipid droplets; ≥25% triglyceride (TG)/ total cholesterol (TC) decrease

^*^This multiphase pipeline captures the key stages of adipogenesis, including commitment, differentiation, and maturation, through well-defined molecular endpoints. *In vitro* assays are integrated with *in vivo* models that evaluate systemic and hepatic lipid metabolism. Collectively, these complementary platforms provide a comprehensive, cross-species assessment of anti-adipogenic efficacy.

One of the leading reasons for late-stage failure in drug development is the combination of suboptimal pharmacokinetic profiles and unexpected toxicities. To mitigate this, we recommend early ADMET profiling: Caco-2 permeability assays (aiming for Papp >10 × 10^−6^ cm/s) to predict oral absorption; human liver microsome clearance studies (targeting moderate intrinsic clearance <30 mL/min/kg); and Cytochrome P450 (CYP450) inhibition panels focusing on CYP3A4, CYP2C9, and CYP2D6 to anticipate drug–drug interactions (Lindmark et al., 2018). Adjustments such as bioisosteric replacement of phenolic hydroxyls with sulfonamide moieties can be explored to enhance metabolic stability without compromising potency.

Beyond the realm of obesity, chromenes have also demonstrated antiviral activity, most notably through inhibition of HIV integrase and protease at low micromolar concentrations (Santiprabhob et al., 2020). Chronic antiretroviral therapy often precipitates lipodystrophy and metabolic syndrome, compounding cardiovascular risk in HIV-infected individuals. This dual anti-adipogenic and antiviral capacity highlights chromene derivatives as promising adjuncts in the management of HIV-related metabolic complications.

A recent study by Perna et al. (2023) demonstrated that metabolic alterations and adipose dysfunction associated with antiretroviral therapy can be partially reversed through compounds that modulate adipocyte lipid metabolism and inflammatory profiles. Although chromenes were not directly tested in their study, the findings underscore the feasibility of targeting adipose tissue dysfunction pharmacologically in the context of HIV-related lipodystrophy, supporting the rationale for evaluating chromene derivatives in similar co-culture models.

We therefore propose co-culture assays in which human preadipocytes differentiated in the presence of protease inhibitors such as lopinavir (Galle et al., 2010) are subsequently treated with lead chromenes, with outcomes evaluated through lipid droplet morphology and adipokine secretion. Ultimately, HIV-infected humanized mouse models should evaluate both viral suppression and adipose tissue health, measuring viral load alongside circulating adiponectin, leptin, and histological markers of adipocyte integrity.

## Discussion

Adipose tissue biology remains central to metabolic disease research, yet drug development often prioritizes pharmacological efficacy over sustainability and translational coherence. Chromene derivatives, owing to their structural versatility and multimodal activity, offer a unique opportunity to bridge these gaps. The framework presented here unites environmental sustainability, mechanistic understanding, and dual therapeutic targeting within a single translational model.

Here, we propose a five-pillar strategy that integrates green chemistry, mechanistic insight, and dual-pathway efficacy to accelerate chromenes from bench to clinic. Standardized green metrics such as Atom Economy, E Factor, and GAPI should be applied early, ensuring synthesis routes are both efficient and environmentally responsible. Integrating these considerations early in the development pipeline may offer a strategic advantage in aligning with emerging standards for environmentally responsible drug development.

From a biological standpoint, capturing the full trajectory of adipogenesis, both *in vitro* and *in vivo*, ensures more accurate assessment of anti-obesity activity. When coupled with rational SAR design, molecular docking, and early ADMET screening, this approach not only sharpens mechanistic clarity but also reduces the risk of late-stage failure.

Beyond metabolic disorders, chromene derivatives exhibit promising antiviral activity, particularly relevant to HIV-associated lipodystrophy, where metabolic dysfunction and viral persistence coexist. The ability of these molecules to modulate adipogenesis while exerting antiviral effects underscores their dual-use potential. Future work should prioritize cross-disciplinary validation—combining computational design, green synthesis, and translational biology—to accelerate safe, sustainable, and clinically relevant chromene candidates.

Finally, the proposed framework moves chromene research beyond isolated findings toward a systematic, environmentally responsible, and mechanistically transparent paradigm. By embedding sustainability into pharmacological innovation, chromenes may serve as a model for the next-generation of small-molecule therapeutics that are both biologically effective and ecologically accountable.
